# Antibiotic effect and microbiome persistence vary along the European seabass gut

**DOI:** 10.1038/s41598-020-66622-5

**Published:** 2020-06-19

**Authors:** Fotini Kokou, Goor Sasson, Itzhak Mizrahi, Avner Cnaani

**Affiliations:** 1 Department of Poultry and Aquaculture, Institute of Animal Sciences, Agricultural Research Organization, Rishon LeZion, Israel; 2 Department of Life Sciences & the National Institute for Biotechnology in the Negev, Ben-Gurion University of the Negev, Beer-Sheva, 84105 Israel; 3 Present Address: Wageningen University and Research, Department of Animal Sciences, Aquaculture and Fisheries Group, Wageningen, Netherlands

**Keywords:** Microbial ecology, Microbiome, Antibiotics, Animal physiology, Genomics

## Abstract

The constant increase in aquaculture production has led to extensive use of antibiotics as a means to prevent and treat diseases, with adverse implications on the environment, animal health and commensal microbes. Gut microbes are important for the host proper functioning, thus evaluating such impacts is highly crucial. Examining the antibiotic impact on gut segments with different physiological roles may provide insight into their effects on these microhabitats. Hence, we evaluated the effect of feed-administrated antibiotics on the composition and metabolic potential of the gut microbiome in the European seabass, an economically important aquaculture species. We used quantitative PCR to measure bacterial copy numbers, and amplicon sequencing of the 16S rRNA gene to describe the composition along the gut, after 7-days administration of two broad-range antibiotic mixtures at two concentrations. While positive correlation was found between antibiotic concentration and bacterial abundance, we showed a differential effect of antibiotics on the composition along the gut, highlighting distinct impacts on these microbial niches. Moreover, we found an increase in abundance of predicted pathways related to antibiotic-resistance. Overall, we show that a high portion of the European seabass gut microbiome persisted, despite the examined antibiotic intake, indicating high stability to perturbations.

## Introduction

The need to sustain and fulfil the protein demand of the increasing world population is leading to an increase in livestock production. Aquaculture is one of the fastest-growing industries, currently reaching an annual production of almost 180 million tons and annual consumption of 21 Kg per capita^1^. Thus, an ever-increasing effort is made to improve both the fish protein yield through an enhanced growth performance and feed efficiency, as well as to boost fish welfare and ability to prosper in the aquaculture environment. To prevent and treat diseases, as well as to improve feed efficiency of livestock animals, there is an extensive application of antibiotics^2^. Within the rapidly growing aquaculture industry, the use of antibiotics is only sparingly regulated, depending on the country. In aquaculture, antibiotics are used either as a feed supplement or occasionally in baths and injections, often even for prophylactic treatment^3,4^.

Beyond the known implications of antibiotic usages, such as the danger of increasing the antibiotic resistance of the microbial environment^2,4–6^, there are also other issues with great importance. Extended antibiotic use may create disturbance in the gut ecosystem, e.g. potential colonization of the ecosystem by pathogenic bacteria due to a reduction in the microbial richness and competition^7,8^. Studies in terrestrial animals have shown that the wide application of antibiotics has a pronounced effect on the microbiome residing in the host gut^9,10^. On the other hand, the effects of antibiotic administration on the fish gut microbiome and the possible outcomes are still under-study^8,11–16^. Since substantial environmental impact can occur through the egestion of aquaculture waste carrying antibiotic-resistant bacteria, the antibiotic effects on the gut microbiome is a focal point from both agricultural and ecological aspect that needs to be further addressed.

To address the impact of antibiotic use on the fish gut microbial communities, we designed a study aiming to evaluate the effects induced by dietary administrated antibiotics along the gut. Different antibiotic mixtures were selected to target a broad range of bacterial groups, using antibiotics previously tested in aquaculture, in regular treatment dosage and in excess, while the mixtures were prepared to include alternatives for each type of antibiotic range group. Examining how microbial communities are affected in specific gut locations with different physiological function may provide significant insight into how antibiotics affect bacterial taxa in these microhabitats. As our experimental model, we used the European seabass (*Dicentrarchus labrax*), one of the most important Mediterranean aquaculture species. Despite its high importance for commercial aquaculture, only limited information exists on its gut microbiota, deriving from the analysis of culturable microorganisms^17^, culture-independent studies using fingerprinting^18,19^ and recently from sequencing technology of the 16S rRNA gene^20–24^. While progress has been made on the culture of this species, the interpretation of its gut microbiome changes remains unclear. Thus, it is important to understand how antibiotic intake may affect microbial communities. In our study, we showed that in-feed antibiotics differently affect the communities and microbial niches along the gut. Despite these changes, our study highlights that European seabass possesses a highly abundant portion of the microbiome, already described in our previous study^22^, with antibiotic persistence. These findings suggest that remarkable stability exists within the fish gut microbiome, but also raise a concern of whether this is an outcome of increased antibiotic resistance in aquaculture facilities.

## Results

### Antibiotic intake effects on bacterial counts across the seabass gut

In our study, we aimed to characterize the effects of in-feed antibiotics on the microbial communities along the gut of the European seabass. The antibiotic concentrations in the feeds were selected according to the recommended concentrations for disease treatments or prevention in farms and aquaculture units (in the range of 0.05–0.1 g kg^−1^ of fish)^25,26^. We used two antibiotic cocktails at two concentration levels: at 5 mg g^−1^ of feed (Low dose), which is the commonly used concentration (corresponding to 0.075 g kg^−1^ of fish); and at 30 mg g^−1^ of feed (High dose; corresponding to 0.45 g kg^−1^ of fish) to also examine the response of the microbial communities in excess of antibiotics. The cocktails were selected among antibiotics previously used in aquaculture^27^ to target a broad range of bacterial groups, while the two mixtures (Mix 1 and Mix 2) were prepared to include alternatives for each type of antibiotic range group (Table 1).

**Table 1:** Antibiotic mixtures used in the present study.

Antibiotic Range	Mix 1	Mix 2
*B-lactam (cell wall synthesis inhibitors)*	Ampicillin^a^	Penicillin^b^
*Aminoglycoside (Protein synthesis inhibitor)*	Kanamycin^c^	Streptomycin^d^
*Macrolides (protein synthesis 50S inhibitor)*	Erythromycin^e^	Lincomycin^f^
*Fluoroquinolones (DNA synthesis inhibitor)*	Ciproflaxine^g^	
*Glycopeptides (Gram positive inhibitor)*	Vancomycin^h^	

^a^Ampicillin anhydrous, 96.0–100.5% (anhydrous basis), A9393-5G.

^b^Penicillin G sodium salt, P3032-100MU.

^c^Kanamycin sulfate from *Streptomyces kanamyceticus*, K4000-5G.

^d^Streptomycin sulfate salt, S9137-25G.

^e^E5389-5G.

^f^Lincomycin hydrochloride, L2774-1MU.

^g^17850-5G-F.

^h^Vancomycin hydrochloride from *Streptomyces orientalis*, V1130-5G.

**Figure 1:**
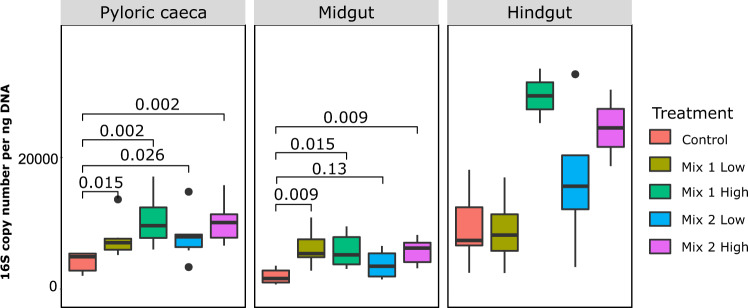
Number of 16S rRNA gene copy numbers measured with quantitative PCR within each gut compartment of fish fed with different diets (control and antibiotics). Significance was tested with Wilcoxon rank-sum two-sided test at P < 0.05.

The common gut of a bony carnivorous fish is composed of three main locations: the pyloric caeca, which are finger-like extensions located in the proximal part of the gut; the midgut, which is the main part of the gut; and the hindgut, the last compartment separated from the midgut by a sphincter^28^. We sampled these three different gut parts of 30 European seabass individuals fed on diets coated with different antibiotic mixtures (n = 6 per diet; Table 1) for a bacterial count and microbial composition analysis (see *Methods section*). Comparison of the 16S rRNA gene copy number showed that the hindgut had significantly higher bacterial counts compared to the rest of the gut (P < 0.05; Fig. 1; Supplementary Fig. 1A), as it was previously reported in fish^29^. Overall, dietary antibiotic intake increased the bacterial counts compared to the control (Fig. 1; Supplementary Fig. 1B), suggesting a higher availability of microbial niches, with potential opportunities for species expansion or invasion.

### Microbial diversity response to in-feed antibiotics

After observing an effect of both gut location (gut part) and antibiotic treatment on the bacterial counts, our next aim was to evaluate these effects on the microbial composition. We used bacterial tag-encoded amplicon sequencing generated from the V4 region of the 16S rRNA gene to identify and characterize the overall fish gut microbial composition in each of our samples. We performed our analysis using DADA2^30^ and after subsampling to an equal number of reads (6394 reads per sample), the overall number of different microbial taxa (Exact Sequence Variant, ESV) detected by the analysis reached 2419, based on 100% nucleotide sequence identity between reads. To assess whether our sampling effort provided sufficient taxa coverage to accurately describe the microbial composition of each group, read-based rarefaction curves were generated (Supplementary Fig. 2), and Good’s coverage index was calculated (Supplementary Table 1). Both analyses implied that our sampling effort was sufficient to characterize the microbial composition in the different gut parts and treatments.

**Figure 2:**
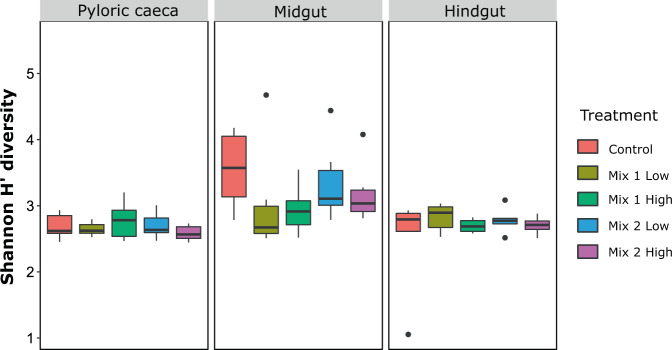
Shannon H’ diversity of the microbial communities of the different diets (control and antibiotics) in each gut part. Significance was tested with Wilcoxon rank-sum two way test at P < 0.05.

Diversity analysis (alpha-diversity) of the microbial communities across the gut revealed significant changes between the different parts (for Shannon H’, Fig. 2 and Supplementary Fig. 3; for Richness, Supplementary Fig. 4). Linear mixed-effects model analysis indicated that gut location was the major factor shaping microbial community diversity and richness within the gut (Table 2; P < 0.001). No significant effect of the antibiotic treatment was found, although a trend for decrease in diversity was observed, mainly in the midgut (Fig. 2).

**Table 2:** Linear mixed-effects model by restricted maximum likelihood (REML) for gut location and treatment on Shannon diversity and richness.

	d.f.	F	*P*-value	Significant contrasts
*Main Effects – Shannon H’ index*				
AIC = 158.89, BIC = 200.12, logLik = −61.44				
Gut location	2	14.80	<0.001*	Midgut higher than pyloric and hindgut
Treatment	4	0.5606	0.06920	
Location: Diet	8	1.5424	0.1591	
*Main Effects – Richness*				
AIC = 715.91, BIC = 757.13, logLik = −339.95				
Gut location	2	12.15	<0.001*	Midgut higher than pyloric and hindgut
Treatment	4	0.51	0.7241	
Location: Diet	8	0.71	0.6737	

*Statistical significance at P < 0.05.

d.f., degrees of freedom; AIC, Akaike information criterion; BIC, Bayesian information criterion; logLik, log of likelihood.

Looking at the beta-diversity, both the gut part and the treatment had a significant impact on the microbial communities (Table 3; Permanova using Bray Curtis metric, *P*_Treatment_ = 0.002, *P*_Part_ = 0.011), as well as the antibiotic concentration (P_Dosage_ = 0.002; Supplementary Table 2). To understand the impact of the antibiotics along the gut, we then further explored their effects on each part separately and observed a differential response. More specifically, when no antibiotics were consumed by the fish (control group), an increase in the bacterial diversity was observed from the pyloric caeca to the midgut, followed by a decrease from the midgut to the hindgut (Supplementary Fig. 3C). This pattern was less evident along the gut of fish fed with the antibiotic mixtures (antibiotic-treated groups), and especially with Mix 1 (Supplementary Figs. 3C; 4D), suggesting that the microbial niches across the gut can be differently affected by antibiotics, potentially through a selection of specific microbial compositions.

**Table 3:** Permanova results for experimental communities based on Bray–Curtis distances.

	d.f.	SS	MS	PseudoF	R^2^	*P*-value
*Antibiotic*	4	0.27876	0.069691	2.12540	0.09209	0.002**
*Gut location*	2	0.14271	0.071354	2.17612	0.04714	0.011*
*Antibiotic x Gut location*	8	0.21200	0.026500	0.80819	0.07003	0.868
*Residuals*	73	2.39365	0.032790		0.79073	
*Total*	87	3.02712			1.00000	

*, **Statistical significance at *P* < 0.05 and 0.01, respectively. Permutations n = 999.

d.f., degrees of freedom; SS, sum of squares; MS, mean sum of squares.

**Figure 3:**
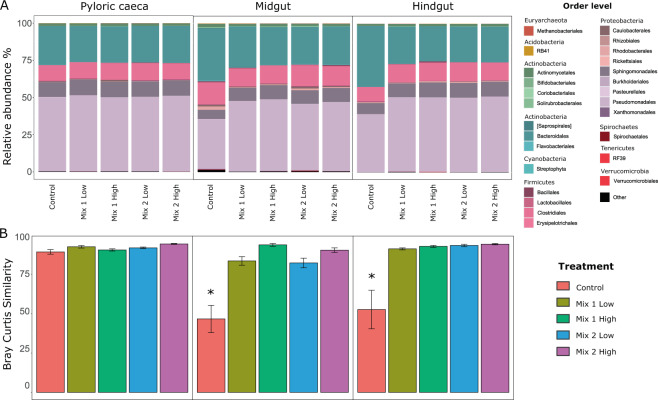
(**A**) Microbial composition at the order level across the gut and within each antibiotic-treated group. (**B**) Bray-Curtis within group similarity across the different gut parts and antibiotic treatments. Stars indicate significance at P < 0.05, after performing Wilcoxon rank-sum two-way test, between the treatments and the control.

To further explore the antibiotic impact on the microbial structure across the gut, we assessed the similarity of the microbial communities (beta-diversity) within each group. Comparison between the control and the antibiotic-treated groups showed a significant effect of the antibiotics on the microbial composition (Fig. 3). More specifically, we found that fish fed with antibiotics had more similar microbial communities (as shown by Bray-Curtis within group similarity) among the individuals within a group (antibiotic treatments and control), or even among the different antibiotic treatments, compared to the control (Supplementary Fig. 5). These results indicate that antibiotics select for specific microbial compositions along the gut, hence, making individual microbiomes look more similar. For example, we observed that specific taxa were enriched after antibiotic intake, mostly from the Proteobacteria phylum, while others were diminished, such as the order Fusobacteria and taxa from the Bacteroidetes phylum (Fig. 3A). Notably, this effect was moderate in the pyloric caeca (Fig. 3B), where the microbial communities were more similar between individuals, regardless of antibiotic intake, suggesting that the physiological conditions in the pyloric caeca allow only specific microbes to exist. Indeed, we measured a lower pH in the pyloric caeca compared to the other intestinal parts (Supplementary Fig. 6), which may have contributed to higher selection pressure in this compartment and thus reducing the antibiotic impact on the microbial communities.

Since we found a significant impact on microbial community structure and composition, our next aim was to further understand the potential effects on the microbial interactions by performing network analysis using Spiec Easi^31^. We hypothesized that microbial composition changes would have an impact in the syntrophic or competitive interactions between microbes^14^. Our network analysis indicated that the ratio of positive to negative edges, indicating interactions, decreased with antibiotic use (Supplementary Fig. 7), suggesting that antibiotic use causes a disruption of syntrophic interactions with potential effects on the stability of microbial communities.

### Functional diversity of the microbiome related to different gut compartments and antibiotic treatment

We showed that the microbiome composition is affected by the gut location and the antibiotic intake, and thus, we evaluated the microbial community attributes in relation to these two factors. Next, we utilized the LEfSe (Linear Discriminant Analysis Effect Size)^32^ and the PICRUSt tool (Phylogenetic Investigation of Communities by Reconstruction of Unobserved States)^33^ to understand the impact of antibiotic intake on the functional diversity of the microbiome. Our findings revealed differences between the pyloric caeca and the midgut regarding metabolic pathways (Supplementary Fig. 8A). More specifically, carbon and glycan metabolism pathways were mostly enriched in the midgut, while amino acid and lipid metabolism pathways were more abundant in the pyloric caeca (Supplementary Fig. 8A), highlighting differences in the microbial community functionality in these two gut parts. Interestingly, we observed an enrichment of genes related to the biosynthesis of the antibiotic gene groups of vancomycin in the midgut, which mainly originated from a higher abundance of these genes in fish fed with Mix 2 (Supplementary Fig. 8B). Although both mixtures contained the antibiotic vancomycin, such results may relate to the absence of a significant decrease in the microbial diversity across the gut in this group (Supplementary Fig. 3C).

**Figure 4:**
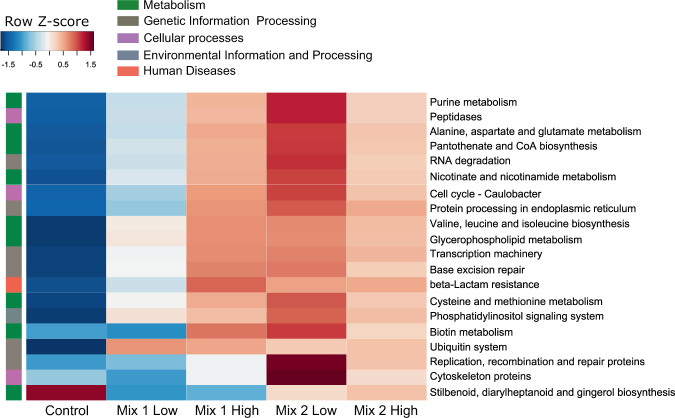
Pathway enrichment analysis using PICRUSt-predicted KEGG (Kyoto Encyclopaedia of Genes and Genomes) orthologs between the antibiotic-fed groups and the control in the midgut. Significance in pathway enrichment was tested using LEfSe analysis.

When we evaluated the impact of antibiotic intake on the functional diversity of the microbiome, we found enrichment of metabolic pathways such as amino acid and cofactors/vitamins metabolism, as well as pathways related to genetic information and processing, like transcription and repair machinery (Fig. 4). Notably, in the antibiotic-treated groups, we observed an increase in antibiotic resistance pathways related to beta-lactam compared to the control group. This increase was associated with an increase in taxa belonging to *Staphylococcus*, *Pseudomonas*, *Janthinobacterium, Bacillus* and *Klebsiella* genera (Supplementary Fig. 9). Such results may indicate that the persistent microbial community species have increased protein repair and replication mechanisms that potentially allow them to survive, while beta-lactam resistance genes seem to be highly prevalent within the fish gut microbial communities, as recently reported in other aquaculture facilities^34^.

### Antibiotic effects on microbial taxa abundance and prevalence

**Figure 5:**
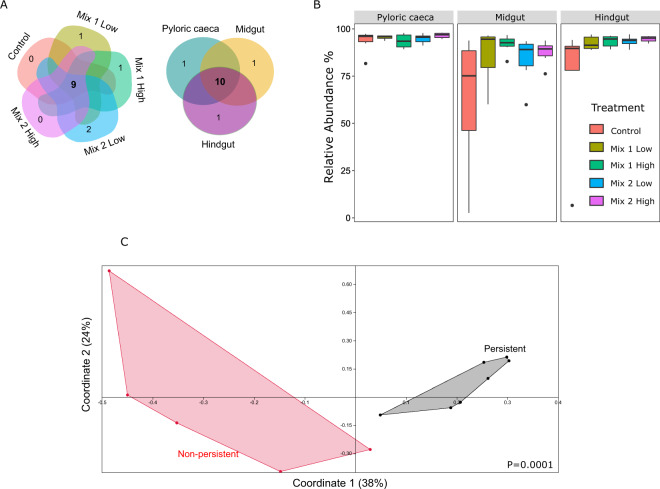
(**A**) The persistent microbiome is shared between treatments (left) and gut parts (right), as indicated by the Venn diagram. (**B**) The relative abundance of the persistent microbiome overall consisted of around 60% of the overall abundance, while it increased with antibiotic resistance, especially observed in the midgut and hindgut. (**C**) Principal Coordinate Analysis based on Jaccard metric on the antibiotic genes’ richness between the persistent and the affected (non-persistent) microbes. P-value (P = 0.0001) indicates significance based on Permanova (Jaccard metric).

Our next aim was to identify the antibiotic effects on the microbial communities, and more specifically, to identify persistent and affected microbiome members. In order to determine persistent microbial communities, we detected taxa that were not affected by antibiotic intake, and thus were shared by at least 80% of the individuals by all treatments (Fig. 5A). We found that most of these taxa were also shared across all gut parts (9 out of 10) and they were highly abundant, occupying an average of 90% of the overall microbial relative abundance (Fig. 5B). Moreover, the abundance of most of these microbes was not negatively affected after antibiotic treatment and in several cases it even increased (Supplementary Fig. 10), suggesting either that they persist to perturbations or that they may even carry antibiotic resistant genes that allow them to survive (Supplementary Fig. 9).

To examine this hypothesis, we investigated the closest bacterial genomes of these persistent microbes, based on sequence identity (Supplementary Table 3), for antibiotic resistance genes. Moreover, in order to compare with taxa that were negatively affected by the antibiotic intake, we also looked into the closest bacterial genomes of microbes that were significantly depleted either by both antibiotic mixtures, or by each one separately (Supplementary Table 4; Supplementary Fig. 11). We found that most of the persistent microbes potentially carry several antibiotic resistance genes (Supplementary Fig. 12), whereas they clustered separately from the affected microbes based on their functional profiles (richness in antibiotic resistant genes; Fig. 5C). Overall, these results suggest that European seabass carries a great portion of the microbiome that is resistant to antibiotics, with members that may survive antibiotic treatment due the presence of some antibiotic resistance genes.

## Discussion

The increase in fish protein consumption over the past years has led to a rapid increase in aquaculture production, and consequently, in the use of antibiotics^3,4,6,35,36^. Despite having some negative impacts, antibiotics have been used to effectively treat bacterial infections in both humans and animals. Antibiotics nowadays are designed to target a broad spectrum of pathogenic populations^37,38^. However, related members of the commensal microbiota can also be affected, leading to marked changes in the microbial community and dysbiosis. Such an application often leads to an expansion of antibiotic-resistant strains and affects intestinal health, since it can eliminate also beneficial microbes which may or may not recover to pre-treatment levels^39^. Moreover, antibiotic administration can negatively affect physiological functions of the host, such as mitochondrial gene expression or enzyme activities^23^. Thus, it is crucial to understand the effects of in-feed antibiotics on the gut microbial communities and to evaluate their impact on gut health. Evaluating and understanding the effects of antibiotics on the composition and metabolic potential of the microbes in different niches along the gut may also provide a better insight on the side effects of antibiotics on the host physiology and performances.

In the present study, we used two antibiotic mixtures in two concentration – one imitating the regular antibiotic use and one in excess – in order to evaluate their impacts on the gut microhabitats of European seabass. In the past years, few studies evaluated the impact of in-feed antibiotics on fish gut, in Atlantic Salmon^14,40^, zebrafish^41^, fathead minnow^16^, channel catfish^8^ and pacu^42^, focusing mainly on single antibiotic and not on mixtures. Several studies also looked into administration of antibiotics through the water, showing significant impacts on both the skin and the gut microbiome^15,43,44^. In the present study, we chose to evaluate different mixtures that were covering a broad range of antibiotic actions and considering that some antibiotics may have synergistic effects^45^ or several bacterial strains can develop resistance^34^, this would allow us to evaluate the impacts of such perturbation on the microbial communities.

### Antibiotic intake increases bacterial counts and affects diversity in seabass gut

Currently, there is limited information to enable an evaluation of the effects of antibiotic use on the fish gut microbiome. The in-feed antibiotic mixtures used in our study, in regularly used levels and excess, drastically affected the bacterial counts across the gut (Fig. 1). As two broad-spectrum antibiotic cocktails were administered in the diets, we expected a wide range of microorganisms to be affected, thus leading to a dramatic change in the microbial richness and diversity. Surprisingly, the overall microbial diversity and richness in the European seabass gut were not significantly affected (Fig. 2), although we observed a trend for the diversity to decrease with antibiotics, mainly in the midgut. Moreover, we found a gradual increase in the bacterial counts using qPCR which correlated with the antibiotic concentration levels. Previous studies in rainbow trout (*Oncorhynchus mykiss*) fed with oxytetracycline, oxolinic acid and sulfaforazole mixtures also showed an increase in the bacterial numbers throughout the gut^46^. Two recent studies in Atlantic salmon^14^ and zebrafish^41^ showed that antibiotic feeding actually increased the microbial richness and diversity. Although in the present study we did not see significant impact on the diversity, we found that bacterial counts increased with antibiotic intake indicating a higher availability of unoccupied niches. Thus, species that were able to survive were also potentially able to occupy these niches and bloom. This hypothesis is also supported by the loss of syntrophic interactions, as indicated by the network analysis between the control and the antibiotic treated groups in the present study (Supplementary Fig. 7), potentially indicating a lower stability and higher susceptibility to intrusions. This evidence supports the current concern that antibiotic treatment can eradicate microorganisms of the normal microbiota, potentially facilitating the proliferation of opportunistic bacteria by reducing competition.

Interestingly, we found that there was a significant impact of the antibiotic concentration on the microbial composition, although not always consistent between the mixtures. It has been previously shown that even low concentrations can have a significant long-term impact on the fish gut microbiome. A study on fathead minnow reported that the impacts of low and high exposure to triclosan antimicrobial compound (10-fold dose difference) did not differ during dietary intake^16^. Such results suggest that even low concentrations can be sufficient to disrupt the microbial communities, potentially leading also to a different recovery rate after exposure. However, this remains to be elucidated, since in the present study we did not follow up on the recovery of the microbial communities after the trial.

### Microbial populations along the gut respond differently to in-feed antibiotics

In order to evaluate the impact of the disturbance to the microbial communities, caused by antibiotic use, it is of great interest to understand these effects on different sections along the gut, where different microbial communities with different functions exist^9^. Indeed, we show that different microbial compositions occur across the European seabass gut, with the highest microbial diversity occurring in the midgut and the lowest in the pyloric caeca (Fig. 2). This comes in agreement with previous studies^22^, although very few of them included the pyloric caeca^47–49^. Such compositions could be the outcome of different physiological conditions^50^, such as pH or nutrient gradients, that can select for specific microbial taxa and thus result in a spatial distribution of the microbiome.

Interestingly, although we did not find a significant antibiotic effect on the overall gut microbiome diversity, we did observe a differential response of the localized gut microbial communities (Figs. 2 and 3), suggesting that the niches across the gut can be affected in a different manner. A previous study in gibel carp (*Carassius auratus gibelio*) revealed effects of antibiotic treatment on the hindgut microbial communities, but not in the proximal part of the gut, while it also reported that the microbial communities across the gut tended to become more similar compared to the non-treated fish^11^. Similar results were also observed in the Atlantic salmon, where the impact of antibiotic treatment was higher in the distal compared to the midgut^14^. Antibiotic administration can also differently affect body sites, as it has been previously shown in European seabass after oxytetracycline dietary administration^23^. In our study, we mostly observed a differential response of the gut microbial diversity in fish fed with Mix 1, while it was less prominent in Mix 2. This suggests that, although antibiotics of similar ranges were used, there was a different impact of these mixtures on the microbial niches, or that the different antibiotics can differ in how broad they are. For example, in those two mixtures erythromycin and lincomycin were used; although these two antibiotics are within the same range of action, inhibiting the synthesis of the 50S ribosomal unit, erythromycin is more commonly used in aquaculture settings and has a broader range than lincomycin^51^.

Nevertheless, when we compared the gut microbial community structure across the gut, we found that individuals fed with the antibiotic mixtures compared to the control group became more similar, irrelevant of the mixture or the dosage. This supports previous claims^11^ that antibiotics tend to make the gut microbial communities more similar to each other by reducing the microbial diversity. Interestingly, the localized impact of the antibiotic intake was only observed in the midgut and hindgut, while no effects were found on the pyloric caeca. As we have previously shown^22^, the pyloric caeca microbial communities are very similar between the individuals (Fig. 3), potentially due to more selective conditions. Thus, we assumed that the communities within the pyloric caeca are adapted to persist^22^, as reflected by the high similarity that was found regardless of antibiotic intake.

### Microbial functional diversity following antibiotic intake is potentially enriched for antibiotic resistance genes and repair mechanisms

Predictions of the functional diversity within the microbial communities across gut sections and antibiotic treatments revealed numerous traits that potentially contribute to the bacterial fitness under these different abiotic conditions. Changes were mostly observed between pyloric caeca and midgut concerning metabolic pathways, while in the hindgut, which is known to be involved mainly with immune and defence mechanisms, we did not observe enrichment of nutrient metabolism pathways. These results reflect the changes in the physiological functions along the intestinal sections^52^. In carnivorous fish, it has been shown that amino acids and lipids are mostly absorbed by the pyloric caeca, as was also suggested by the results from our enrichment analysis, while larger molecules, such as carbohydrate, are absorbed by more distal areas of the gut^53^.

One of the main concerns from the wide use of antibiotic in livestock and aquaculture is the elimination of beneficial microbiota and the increase of either opportunistic and/or antibiotic resistant taxa. In our setup, we observed an overall enrichment of pathways related to biosynthesis of vancomycin groups, mostly originating from the group fed with Mix 2 in the midgut (Fig. 2; Supplementary Fig. 8B). Moreover, an increase in pathways related to beta-lactam resistance was found in all antibiotic-treated fish, and mostly in the group fed with high levels of Mix 1 (Fig. 4). It has been recently reported that the gut microbiome of Rohu (*Labeo rohita*) is highly abundant in beta-lactam resistance genes (90% of the overall antibiotic resistant genes)^34^. Moreover, oral antibiotics in *Piaractus mesopotamicus* were reported to increase antibiotic resistant genes in the gut^42^.

Considering the increased bacterial counts and changes related to the microbial diversity across the gut in the antibiotic-fed fish in our study, we hypothesize that the surviving microbial species carried antibiotic resistance genes that enable them to increase their abundance. As both of our antibiotic cocktails contained beta-lactam (ampicillin and penicillin, respectively), such an increase could be related to the increase of microbial taxa with resistance to these antibiotics, as observed also in a study with medicated pigs^10^. Indeed, we observed that the increase of the beta-lactam resistance genes was associated with an increase in *Staphylococcus* species as well as *Pseudomonas* species, while overall the increase was associated with an increase in Proteobacteria (Supplementary Fig. 9). Antibiotic resistance have been reported for *Staphylococcus* species^54^, while species of this genus have been reported to be associated with diseased cyprinids^55^. Although our analysis is based on metagenomic predictions based on closest-genome references, the potential increase in antibiotic resistance genes within aquaculture settings should be further explored in the future using metagenomic analysis.

### European seabass possesses an abundant subset of the gut microbiome with antibiotic persistence

In the present study, we found nine abundant members of the gut microbiome that were persistent across treatment and gut parts, some of them being previously reported as part of the European seabass core microbiome^22^. Such microbiomes with high resistance to perturbation related to aquaculture practices have been previously reported in rainbow trout^56^, while high resilience of dominant microbes to antibiotic treatment has been also previously reported in humans^57^. Moreover, the microbes’ overall abundance in the present study increased with the antibiotic treatment (from ~60% in the control, to ~90% in the antibiotic-treated groups). Interestingly, some of these species, like the *Pseudomonas* and *Aeromonas* species, were also identified to increase in the channel catfish (*Ictalurus punctatus*) gut microbiome after florfenicol intake^8^, while they were also part of the gill and skin European seabass microbiome^23^. Most of these genera belong to the phylum Proteobacteria, one of the most abundant phyla in the fish gut, which has been also recently reported to increase after antibiotic feeding in salmon gut^14^. Our analysis, as indicated by the closest genomes’ evaluation of these species, showed that these taxa may carry various sets of antibiotic resistant genes (Fig. 5), with some of them potentially contribute to beta-lactam resistance (Supplementary Fig. 9). Nevertheless, the fact that functions such as pathways related to transcription machinery, base excision repair, protein processing and repair were enriched due to antibiotic treatment indicates that a potential machinery was available to enable the survival of these resistant species^58,59^. To verify those findings, further studies are required using metagenomic information in order to understand better the impacts of such treatments on antibiotic resistance and bacteria adaptation mechanisms.

## Conclusions

To conclude, in the present study we showed that microbial niches along the gut of European seabass were differently affected by antibiotic treatments. Both low and high dosage showed a negative effect on the gut microbial diversity and composition. However, we found a highly abundant portion of the microbiome with antibiotic persistence. Whether this comes from an increase in the number of antibiotic resistant genes within aquaculture settings or a remarkable stability of the microbiome to perturbations still remains to be elucidated. Despite that, a highly abundant microbiome may inhibit invasion of new species into the gut and their ability to establish a stable population. Further studies are required to understand the microbiome resilience, the implications on the host metabolism, as well as the forces that shaped and led to this convergent microbiome composition. Pre-exposure of the animals to stressful conditions may also be a factor that could increase the microbiome resistance to subsequent perturbations, since the fish used in the present trial were gradually transferred to a low-salinity environment during early stage. Ability to assess and quantify the effects of antibiotics, the mode of administration (in-feed or water) on the microbiome, as well as the recovery outcome, will contribute in a better understanding on their impacts on fish fitness, immunity and production indices. Although in the current study we only looked into impact on the gut microbial communities, assessment of such impacts on the gills and skin is also important for future studies, since these communities contribute as the first barriers to pathogenic invasions.

## Methods

### Experimental design and sampling

This study was approved by the Agricultural Research Organization Committee for Ethics in Using Experimental Animals and was carried out in compliance with the current laws governing biological research in Israel (Approval number: 489/14). In this study, European seabass (*Dicentrarchus labrax*) juveniles were obtained from a commercial hatchery (ARDAG Hatchery, Eilat, Israel) and housed in 250-L experimental indoor tanks equipped with recirculating systems, where they were acclimated to experimental conditions. Prior to the experiment, 90 fish with an average weight of approximately 37 ± 7 g were distributed randomly and evenly in 15 experimental tanks. Fish were fed to satiation twice a day (9:00 and 14:00) with a commercial extruded diet (dry pellets, 56% crude protein and 14% crude fat, “Raanan Fish Food”, Israel). The light source was natural photoperiod, providing a light intensity of 1,200 lx during the day. The water was heated and maintained at 25 ± 0.5 °C, using submersible aquarium heaters. After a week of acclimatization in salinity of 25 ppt, fish were slowly acclimated to a low salinity of 3 ppt, over a period of 72 h, which was kept till the end of the experiment^60^. European seabass is an euryhaline species, with a high resilience to salinity changes, especially during its juvenile stage. In the natural environment, juveniles migrate from seawater to lagoons and estuaries where salinity is lower than the seawater. In aquaculture practices, it is common to culture European seabass in freshwater. The reason is that it allows rearing of this species in ponds and recirculating systems (used also in the present study). In these systems, freshwater is preferable since it provides benefits compared to seawater in terms of maintenance and water quality^61^. In this experiment, the fish were gradually acclimatized in low salinity conditions, without being greatly impacted by the salinity changes^60^. Ammonium, nitrite and nitrate were monitored three times per week throughout the trial.

Five different treatment groups of fish were formed in triplicates: a group fed with the commercial diet (control group) and four groups fed with the commercial diets coated with two different mixtures of antibiotics of different range (Sigma Aldrich, St. Louis, MO), at concentrations 5 mg gr^−1^ of feed and 30 mg gr^−1^ of feed (Table 1). Gelatin was used to coat the antibiotic mixtures on the pellets and the pellets were well homogenized and dried overnight in room temperature. The fish were fed at 1% of the body mass twice daily (9:00 and 14:00) for 7 days. At the end of the experiment, three fish from each tank were randomly selected and their guts were dissected using sterile instruments and separated into pyloric caeca, midgut and hindgut. After dissection, each sample was ground, frozen and stored at −80 °C for further analysis. Sampling was performed after an overnight fasting period.

### DNA extraction

Bacterial DNA was isolated from gut samples using the protocol described by Roeselers *et al*.^62^ with some modifications^19,22^. The gut samples were placed in 2.0 ml screw-cap tubes with 0.5 mm and 1 mm silica beads, 400 ml 50 mM Na-phosphate buffer (pH 8.0) and 200 ml of lysis solution containing 5% w/v sodium dodecyl sulfate, 0.5 M Tris-HCl (pH 8.0) and 0.1 M NaCl. A bead-beater was used to homogenize the samples for 5 min on high speed and centrifuged at 16,000 g for 5 min. The supernatant was transferred to new tubes and lysozyme (Sigma, St. Louis, MO) was added to a final concentration of 2 mg/ml followed by incubation at 42 °C for 1 hr and then at 37 °C for 1 hr. Following this step, the solution was sequentially extracted with TE [10 mM Tris-HCl (pH 8.0) and 1 mM EDTA], saturated phenol, phenol-chloroform (1:1 v/v), and chloroform-isoamyl alcohol (24:1 v/v). To collect the DNA in the aqueous phase, precipitation was performed with 0.1 volume of 3 M sodium acetate (pH 5.2) and 0.7 volume isopropanol. The concentration of DNA in the solution was measured using a Nanodrop 2000 UV-Vis spectrophotometer (Thermo Scientific) and DNA was stored at −20 °C for further analysis. Only samples that resulted in a high yield of high-quality DNA were used for subsequent analyses.

### Real time PCR

Quantitative real-time PCR (qPCR) analysis was performed to investigate the relative abundance of bacteria inside each intestinal sample through amplification of their copy of the 16S rRNA gene^22^. Real-time PCR was performed in a 10-ul reaction mixture containing 5 ul Absolute Blue SYBR Green Master Mix (Thermo Scientific), 0.5 ul of each primer (10 mM working concentration), 3 ul nuclease-free water and 2 ul of 100 ng DNA templates. The primers used, targeting the V3 region of the 16S rRNA gene^63^, were: Forward 5′-ACTCCTACGGGAGGCAGC-3′ and Reverse 5′-GTATTACCGCGGCTGCTGGCA-3′). Amplification involved one cycle held at 95  °C for 15 min for initial denaturation and activation of the hot-start polymerase system, and then 40 cycles at 95 °C for 10 sec followed by annealing for 15 sec at 60 °C and extension at 72 °C for 20 sec. Quantification of the bacteria was performed using a standard curve for the 16S rRNA gene in different concentrations (10^2^–10^8^ copies/ul) and the results were expressed as 16S copy numbers per ul.

### Sequencing of gut microbiome

Sequencing of the PCR-amplified V4 region of 16S rRNA, using primers 515 F (5′-GTGCCAGCMGCCGCGGTAA-3′) and 806 R (each R contained a different 12-bp index), was performed using a MiSeq2000 Next Generation system (Illumina), as described in our previous manuscript^48^. First, amplification of the V4 region was performed and the PCR product was quantified using a standard curve with serial DNA concentrations (0.1–10 nM). Finally, the samples were equimolarly diluted to a concentration of 0.4 nM and prepared for sequencing according to the manufacturer’s instructions. An open-source software package, DADA2^30^, was applied to model and correct Illumina-sequenced amplicon errors. Data were demultiplexed into forward and reverse reads according the barcode sequence into sample identity, and trimming was performed. For the forward reads and based on the quality profiles, the first 140 nucleotides were kept and the rest were trimmed, while for the reverse reads, the last 150 nucleotides were kept. DADA2 resolves differences at the single-nucleotide level and the end product is an amplicon sequence variant table, recording the number of times each exact sequence variant (ESV) was observed in each sample (100% sequence identity). Taxonomy was assigned using the Ribosomal Database Project Classifier^64^ against the 16S gene reference Greengenes database (13.8 version)^65^. Due to variation of sequence depths between samples, all samples were normalized to the lowest depth by subsampling (6394 read/sample).

### Comparison of gut communities

For the alpha-diversity analysis, Shannon H’ diversity and richness (observed taxa) were calculated. Cluster analyses exploring the similarities between gut community compositions of different samples were examined using ESV abundance (Bray-Curtis metric). The examination of differentially abundant ESVs between the non-treated (control) and antibiotic-treated groups was performed using DESeq2 tool.

### Functional profile of the gut microbiome

In order to predict the functional content of the gut microbiome originating from the different groups, we used the PICRUSt tool (Phylogenetic Investigation of Communities by Reconstruction of Unobserved States; PICRUSt^33^. The paired-end merged 16S sequences were used for closed-reference OTU picking using QIIME^66^. For this purpose, we re-analyzed our sequences, as described before^59^, using the QIIME close reference protocol against the 97% similarity to the GreenGenes database^65^. The resulting OTU table was then imported into PICRUSt and functional predictions were made according to the metagenome inference workflow described by the developers. The Nearest Sequenced Taxon Index (NSTI) score was used to evaluate the availability of reference genomes that are closely related to the most abundant microorganisms in the samples. High scores (>0.15) generally mean few related references are available and predictions will be of low quality. Low scores (roughly < 0.06) indicate availability of closely related reference genomes^33^. In the present study, an average NSTI score of 0.05 was found (Supplementary Table 5), suggesting that our dataset was closely related to the reference genomes. PICRUSt results were normalized, and then analysed using the LDA Effect Size (LEfSe)^32^.

### Shared and unique microbiome

Analysis of core species was conducted based on the ESV table generated by DADA2, using R software^67^. The shared species were defined as those that were present in at least 80% of the samples for either each fish gut part or each treatment.

### Statistical analysis

Non-parametric Kruskal–Wallis one-way ANOVA and the Wilcoxon t-test were used to test whether the means and standard deviations of real time PCR were significantly different (*P* < 0.05). Non-parametric tests (Wilcoxon test) and linear mixed-effect models (nlme R package^68^) were used to assess α-diversity, while Adonis implementation of Permanova (vegan R package^69^) was used for comparison between groups for β-diversity analysis using Bray–Curtis distance matrix (evaluating both abundance and presence-absence of ESV in each sample). Similarity of gut microbial compositions were evaluated within samples of a group and between different groups using non-parametric t-test using 1000 Monte Carlo permutations. Exact sequent variants that had a differential abundance between antibiotic treatments and control were detected using Deseq2 tool^70^. LEfSe^32^ was used to estimate the effect size of each differentially abundant PICRUSt feature and to perform dimension reduction using the Galaxy online tool (huttenhower.sph.harvard.edu/galaxy/). Moreover, network construction was performed using Spiec Easi^31^ between the different treatment groups, to assess the microbial co-occurrence relationships between microbes, using the recommended parameters. Significance was assessed by rarefying the table, using subsampling at 4000 reads sequence depth, and performing the analysis for each group using Mann-Whitney test.

## Supplementary information

Supplementary Information.

## Data Availability

Sequencing data can be found at the NCBI (SRA) database under the study accession code SRP216741.
