# Supplementary material for: Antibiotic effect and microbiome persistence vary along the European seabass gut
Source: Sci Rep. 2020 Jun 19;10:10003. doi: 10.1038/s41598-020-66622-5 (PMC7305304; doi:10.1038/s41598-020-66622-5)
Supplement: Supplementary file 1 — Supplementary Information. [file 41598_2020_66622_MOESM1_ESM.pdf]

Supplementary Information

Antibiotic effect and microbiome persistence vary along the European seabass gut

Fotini Kokou[1,2,3*], Goor Sasson[2], Itzhak Mizrahi[2], Avner Cnaani[1*]

[1]Department of Poultry and Aquaculture, Institute of Animal Sciences, Agricultural Research Organization, Rishon Letziyon, Israel

[2]Department of Life Sciences & the National Institute for Biotechnology in the Negev, Ben-Gurion University of the Negev, Beer-Sheva 84105, Israel

[3]Wageningen University and Research, Department of Animal Sciences, Aquaculture and Fisheries Group, Wageningen, Netherlands

*Corresponding authors: Fotini Kokou, fotini.kokou@wur.nl, Wageningen University and Research, Aquaculture and Fisheries Group, Netherlands; Avner Cnaani, avnerc@agri.gov.il, Department of Poultry and Aquaculture, Agricultural Research Organization, Israel

[3]*Present address*

Supplementary Tables

Supplementary Table 1 | Good's coverage across the samples.

Control	Pyloric caeca	0.999
	Midgut	1.000
	Hindgut	0.999
Mix 1 Low	Pyloric caeca	0.999
	Midgut	1.000
	Hindgut	0.999
Mix 1 High	Pyloric caeca	0.999
	Midgut	0.999
	Hindgut	0.999
Mix 2 Low	Pyloric caeca	0.999
	Midgut	0.999
	Hindgut	0.999
Mix 2 High	Pyloric caeca	0.999
	Midgut	0.999
	Hindgut	0.999

Supplementary Table 2 | Permanova results for experimental communities based on Bray–Curtis distances.

	**d.f.**	**SS**	**MS**	**PseudoF**	***P*-value**
Dosage	2	0.18297	0.182970	5.8113	0.002**
Part	2	0.14322	0.071612	2.2745	0.006**
Antibiotic x Dosage	*4*	0.11916	0.059580	1.8923	0.077
Residuals	79	2.58177	0.031485		
Total	87	3.02712			

*, **Statistical significance at $P < 0.05$ and 0.01, respectively. Permutations n = 999.

d.f., degrees of freedom; SS, sum of squares; MS, mean sum of squares.

Supplementary Table 3 | Bacterial genome NCBI IDs used for functional analysis in RAST and percentage similarity with the persistent microbes.

ESV ID – taxonomy	Closest bacterial species	% Similarity
Pseudomonas veronii	*Pseudomonas veronii R02*	100
Janthinobacterium lividum	*Janthinobacterium lividum PAMC 25724*	100
Acinetobacter sp.	*Acinetobacter junii strain 65*	100
Pseudomonas fragi	*Pseudomonas fragi NBRC 3458*	100
Stenotrophomonas sp.	*Stenotrophomonas maltophilia ATCC 13637*	99.2
Comamonadaceae unknown sp.	*Limnohabitans planktonicus II-D5*	99.6
Aeromonadaceae sp.	*Aeromonas hydrophila ATCC 7966*	100

Supplementary Table 4 | Bacterial taxa that were significantly depleted from antibiotic intake and the genome of their closest relatives used for functional analysis in RAST.

ESV taxonomy	Closest bacterial species	% Similarity	Affected Mixture
Ruminococcaceae	*Anaerotruncus rubiinfantis M15*	100	Mix 1 and 2
Porphyromonas endodontalis	*Porphyromonas endodontalis ATCC 35406*	96.5	Mix 1 and 2, High
Paraprevotellaceae unknown sp.	*Prevotella buccae ATCC 33574*	84.5	Mix 1 and 2, High
Prevotella sp.	*Prevotella ruminicola 23*	96.0	Mix 1 and 2
Coprobacillus sp	*Coprobacillus cateniformis JCM10604*	91.0	Mix 1 and 2, High
Plesiomonas shigelloides	*Plesiomonas shigelloides 302-73*	100	Mix 1 and 2, High

Supplementary Table 5 | Nearest Sequenced Taxon Index (NSTI) of each sample, showing how closely related are the microorganisms within each sample to the reference genomes used for the predictions. The lowest the score is (<0.06) the better the prediction. The average score was 0.04 in our dataset. M1_5: Mix 1 low; M1_30:Mix 1 High; M2_5: Mix 2 Low; M2_30: Mix 2 High

Sample	Part	Treatment	Value
80	Pyloric caeca	Control	0.040301
81	Pyloric caeca	M1_5	0.039983
82	Pyloric caeca	M1_30	0.040518
83	Pyloric caeca	M2_5	0.042462
84	Pyloric caeca	M2_30	0.042129
85	Pyloric caeca	Control	0.041038
86	Pyloric caeca	M1_5	0.041782
87	Pyloric caeca	M1_30	0.039677
88	Pyloric caeca	M2_5	0.053526
89	Pyloric caeca	M2_30	0.038751
90	Pyloric caeca	Control	0.046132
91	Pyloric caeca	M1_5	0.040109
92	Pyloric caeca	M1_30	0.040561
93	Pyloric caeca	M2_5	0.040232
94	Pyloric caeca	M2_30	0.038446
95	Pyloric caeca	Control	0.040233
96	Pyloric caeca	M1_5	0.042979
97	Pyloric caeca	M1_30	0.03969
98	Pyloric caeca	M2_5	0.040477
99	Pyloric caeca	M2_30	0.040316
100	Pyloric caeca	Control	0.045549
101	Pyloric caeca	M1_5	0.045183
102	Pyloric caeca	M1_30	0.039226
103	Pyloric caeca	M2_5	0.040444
104	Pyloric caeca	M2_30	0.041658
105	Pyloric caeca	Control	0.041008
106	Pyloric caeca	M1_5	0.041397
107	Pyloric caeca	M1_30	0.050427
108	Pyloric caeca	M2_5	0.040037
109	Pyloric caeca	M2_30	0.041078
113	Midgut	Control	0.068398
114	Midgut	M1_5	0.039437
115	Midgut	M1_30	0.037848
116	Midgut	M2_5	0.048983
117	Midgut	M2_30	0.042021
118	Midgut	Control	0.047204
119	Midgut	M1_5	0.0419
120	Midgut	M1_30	0.046077
121	Midgut	M2_5	0.042896
122	Midgut	M2_30	0.042169
123	Midgut	Control	0.042203
124	Midgut	M1_5	0.040757
125	Midgut	M1_30	0.043899
126	Midgut	M2_5	0.041387

127	Midgut	M2_30	0.052143
128	Midgut	Control	0.047428
129	Midgut	M1_5	0.040418
130	Midgut	M1_30	0.051807
131	Midgut	M2_5	0.044146
132	Midgut	M2_30	0.044112
133	Midgut	Control	0.066961
134	Midgut	M1_5	0.039683
135	Midgut	M1_30	0.043541
136	Midgut	M2_5	0.041179
137	Midgut	M2_30	0.041131
138	Midgut	Control	0.048562
139	Midgut	M1_5	0.064558
140	Midgut	M1_30	0.041449
141	Midgut	M2_5	0.06749
142	Midgut	M2_30	0.052817
146	Hindgut	Control	0.039717
147	Hindgut	M1_5	0.038614
148	Hindgut	M1_30	0.039756
149	Hindgut	M2_5	0.040347
150	Hindgut	M2_30	0.038893
151	Hindgut	Control	0.039647
152	Hindgut	M1_5	0.051041
153	Hindgut	M1_30	0.039381
154	Hindgut	M2_5	0.039325
155	Hindgut	M2_30	0.040038
156	Hindgut	Control	0.042584
157	Hindgut	M1_5	0.04442
158	Hindgut	M1_30	0.041562
159	Hindgut	M2_5	0.041194
160	Hindgut	M2_30	0.041106
161	Hindgut	Control	0.039933
162	Hindgut	M1_5	0.039672
163	Hindgut	M1_30	0.039403
164	Hindgut	M2_30	0.041218
165	Hindgut	Control	0.089766
166	Hindgut	M1_5	0.04303
167	Hindgut	M1_30	0.040243
168	Hindgut	M2_5	0.039672
169	Hindgut	M2_30	0.039723
170	Hindgut	M1_5	0.039867
171	Hindgut	M1_30	0.045891
172	Hindgut	M2_5	0.044051
173	Hindgut	M2_30	0.04134

Supplementary Figures

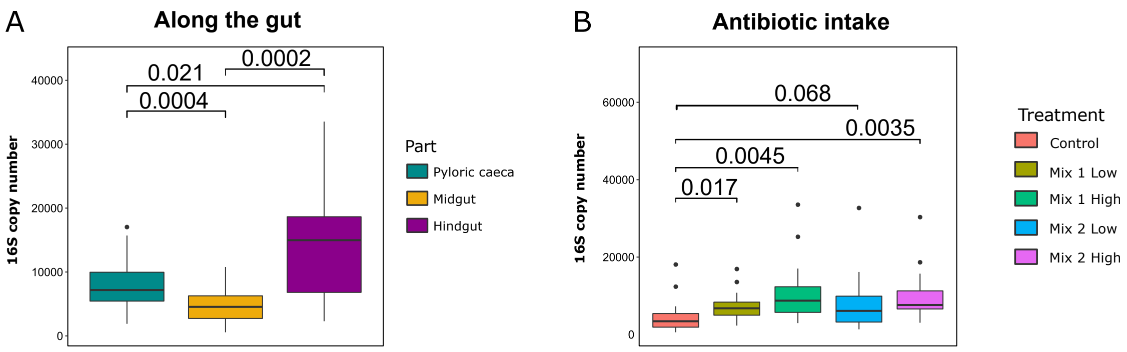

Supplementary Figure 1. Number of 16S rRNA gene copy numbers measured with quantitative PCR. **A.** In the different gut compartments, pyloric caeca, midgut and hindgut. **B.** In the antibiotic-treated groups and the control. Significance was tested with Wilcoxon rank-sum two-sided test at $P<0.05$.

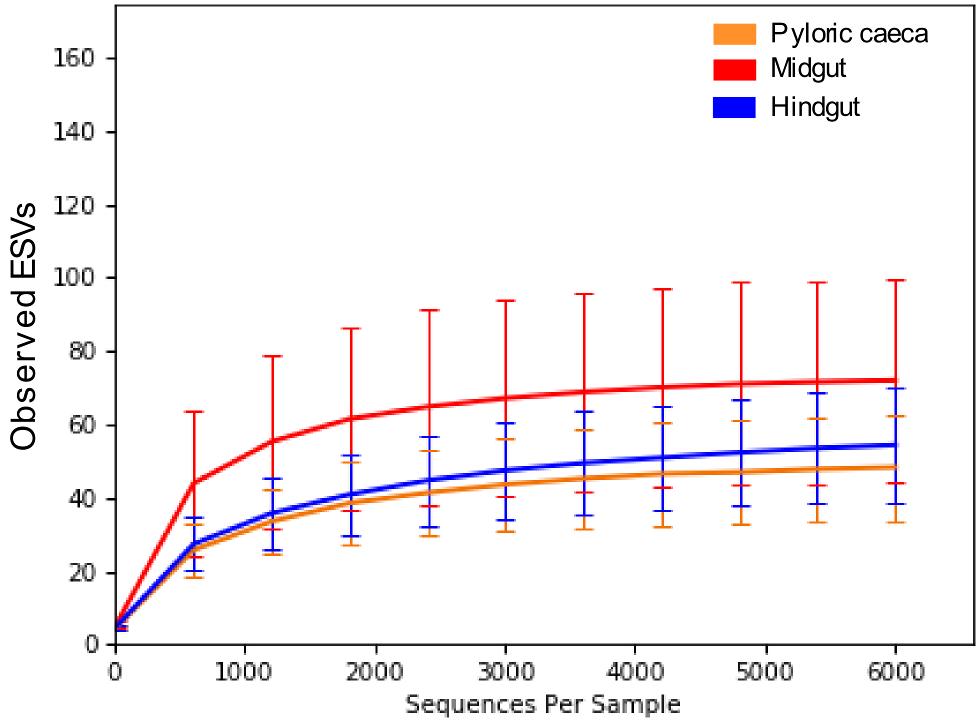

Supplementary Figure 2. Rarefaction curves per sequence depth in the different gut parts.

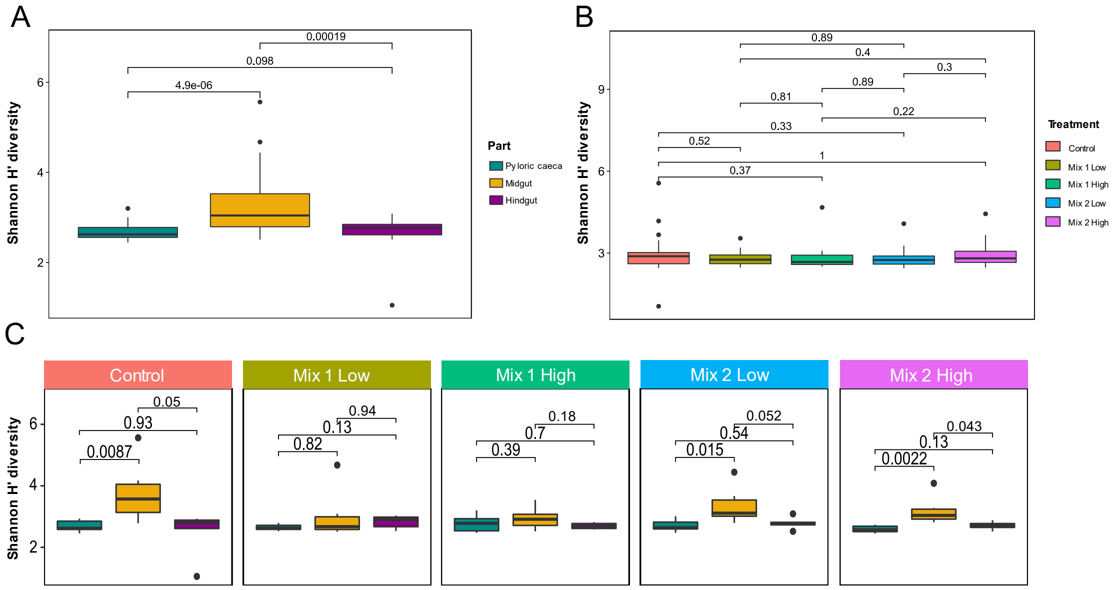

Supplementary Figure 3. Shannon H' diversity of the microbial communities, **A.** within each gut part, **B.** across antibiotic-treated groups and control, **C.** across gut parts within each antibiotic-treated group and control. Significance was tested with Wilcoxon rank-sum two-way test at P<0.05.

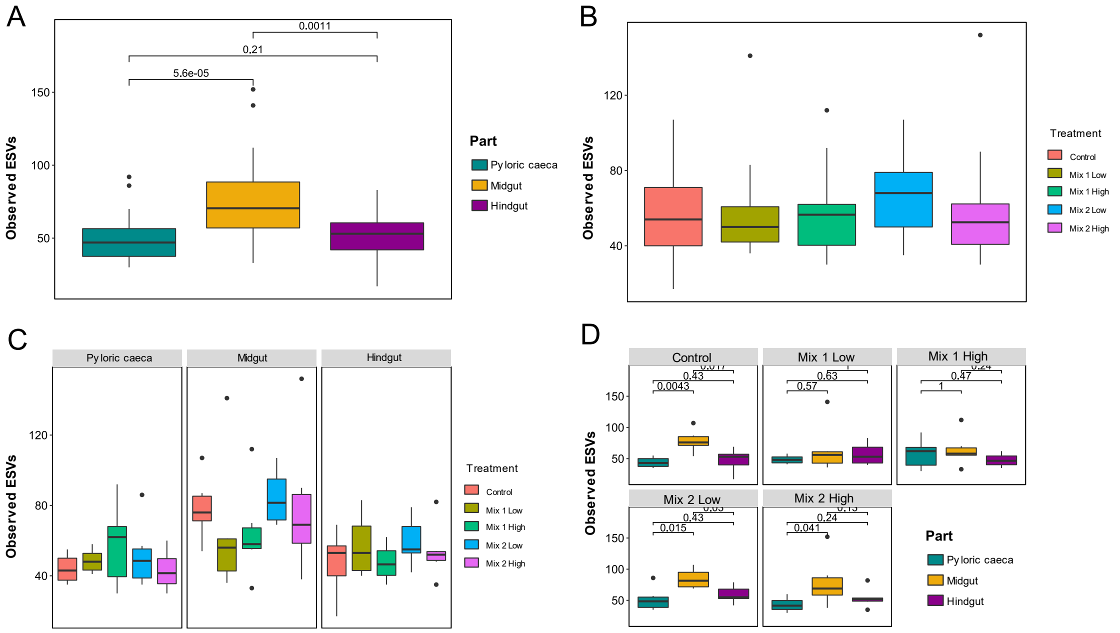

Supplementary Figure 4. Richness of the microbial communities **A.** within each gut part**, B.** across antibiotic treatment, **C.** within each gut across antibiotic-treated groups and control, and **D.** across gut parts within each antibiotic-treated group and control. Significance was tested with Wilcoxon rank-sum two-way test at P<0.05.

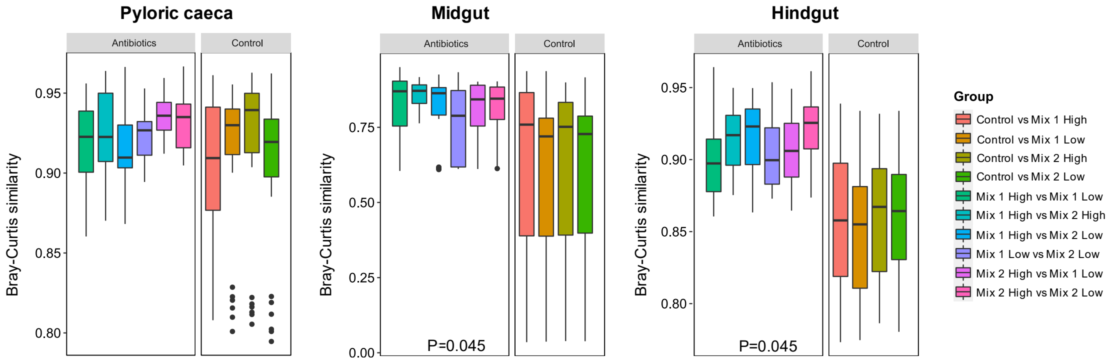

Supplementary Figure 5. Between-group similarity of control and antibiotic-treated groups, showing slightly significant lower similarity between the treated and control groups using Bray-Curtis metric in the midgut and hindgut. P-values indicate the difference between control to antibiotics (right) vs. between antibiotics comparisons (left).

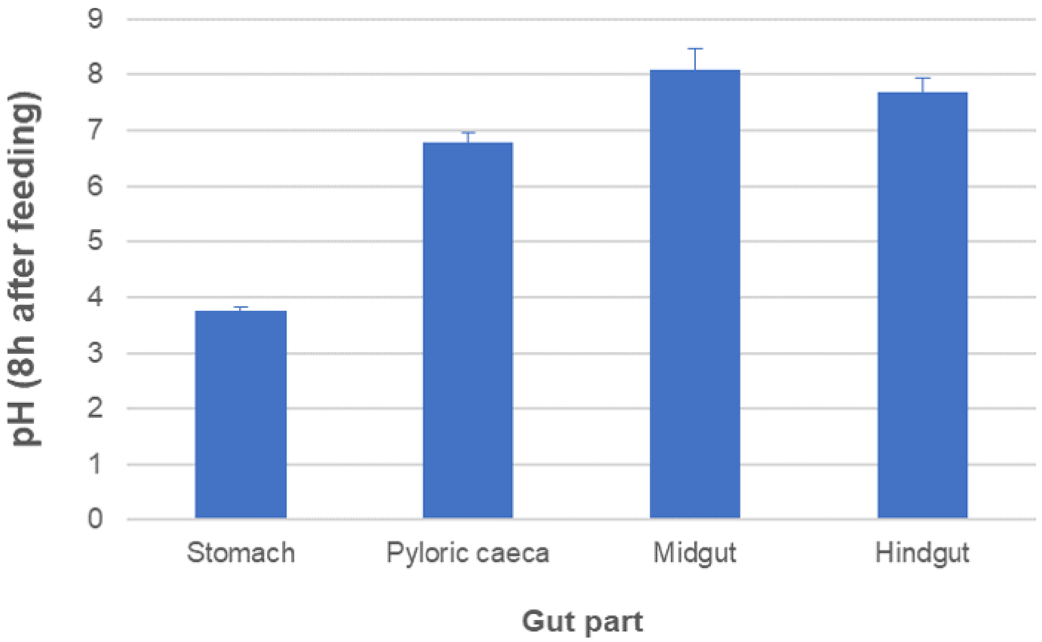

Supplementary Figure 6. Mean values of pH in the different gut parts 8h after feeding. Measurements obtained from Nikolopoulou et al. (2011) and our measurements in the group in seabass individuals (n=5), using a micro-pH meter. (Data obtained from Kokou et al., 2019).

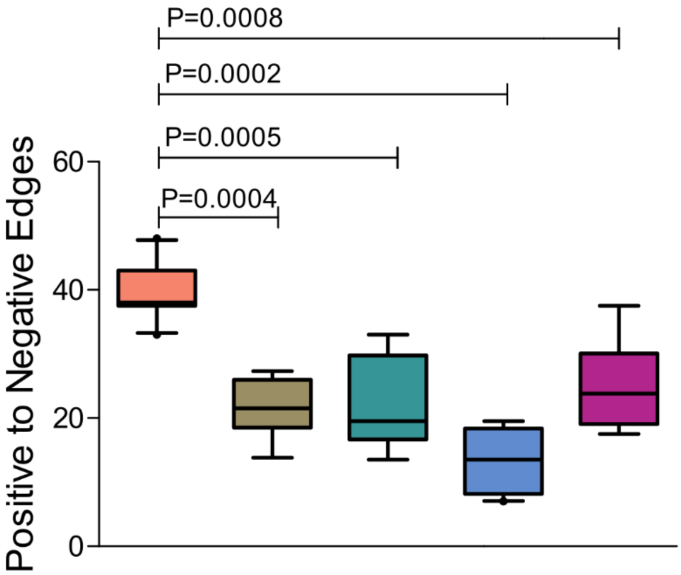

Supplementary Figure 7. Network analysis using Spiec Easi, showing the positive to negative edges ratio. Significance was tested with Wilcoxon rank-sum two-way test at level $P<0.05$.

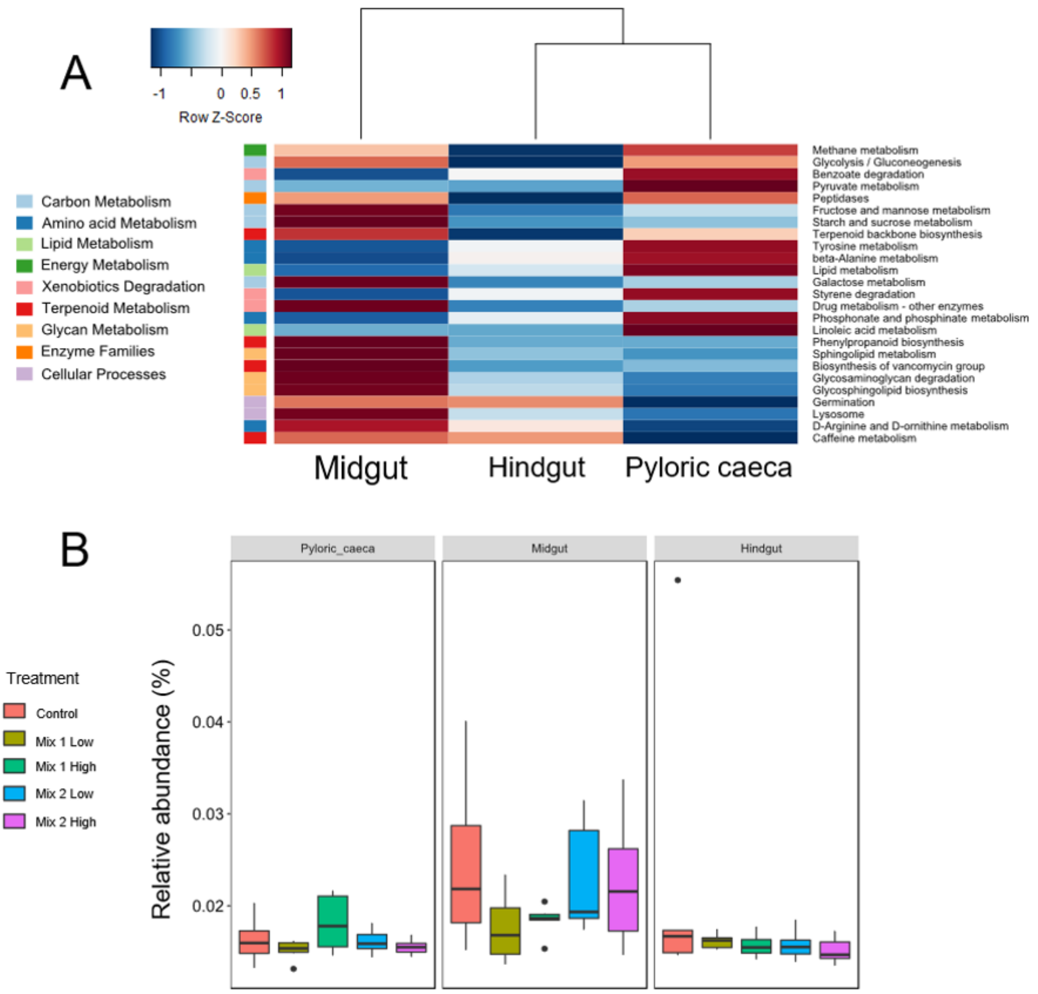

Supplementary Figure 8. A. Pathway enrichment analysis using PICRUSt-predicted KEGG (Kyoto Encyclopedia of Genes and Genomes) orthologs between the gut parts. Different pathways enrichment was indicated by the LEfSe analysis. **B.** Relative abundance of genes in the biosynthesis of vancomycin group pathway across the gut parts and antibiotic-treated groups.

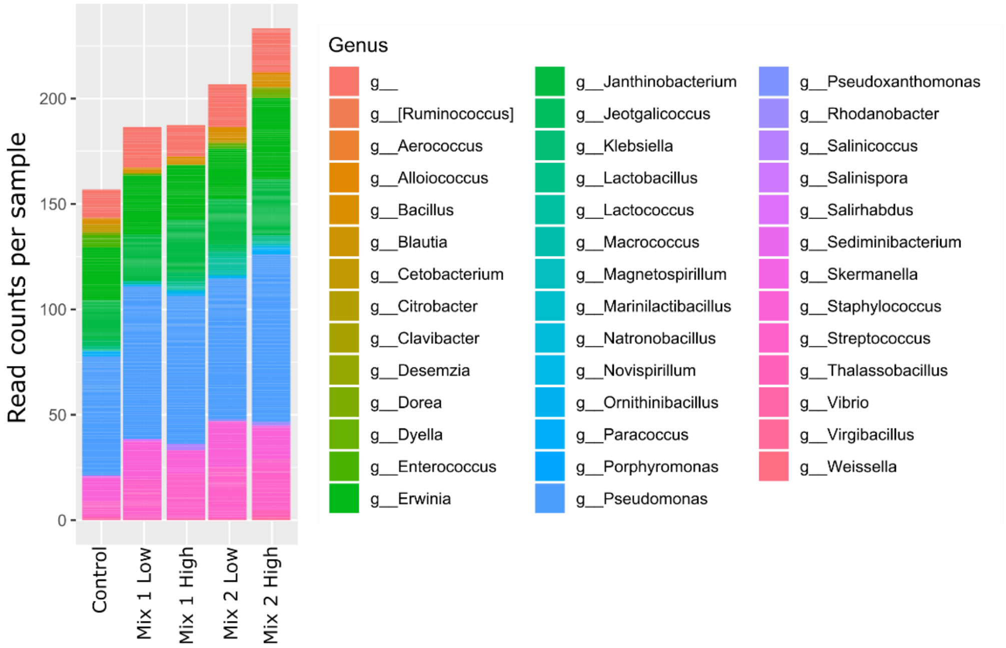

Supplementary Figure 9. Taxa contributing to enrichment of beta-lactam resistance, based on PICRUSt analysis. The taxa are presented at the genus level, showing their average read count in the samples per treatment.

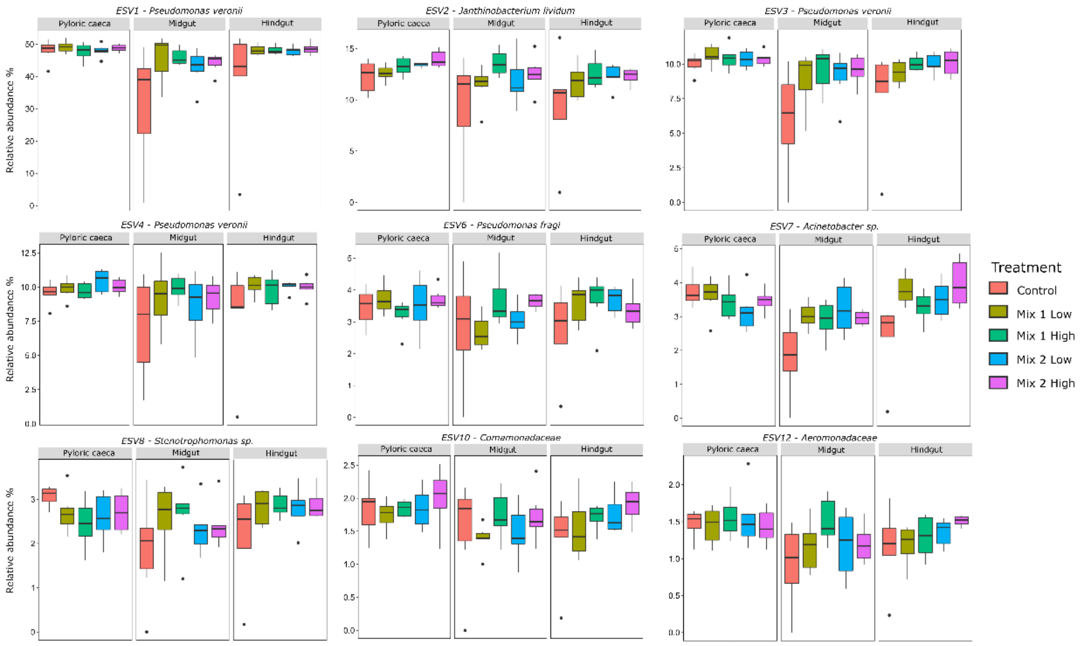

Supplementary Figure 10. Relative abundance of persistent taxa across parts and diets. On the Y-axis is shown the relative abundance in % and on the X-axis the different dietary groups.

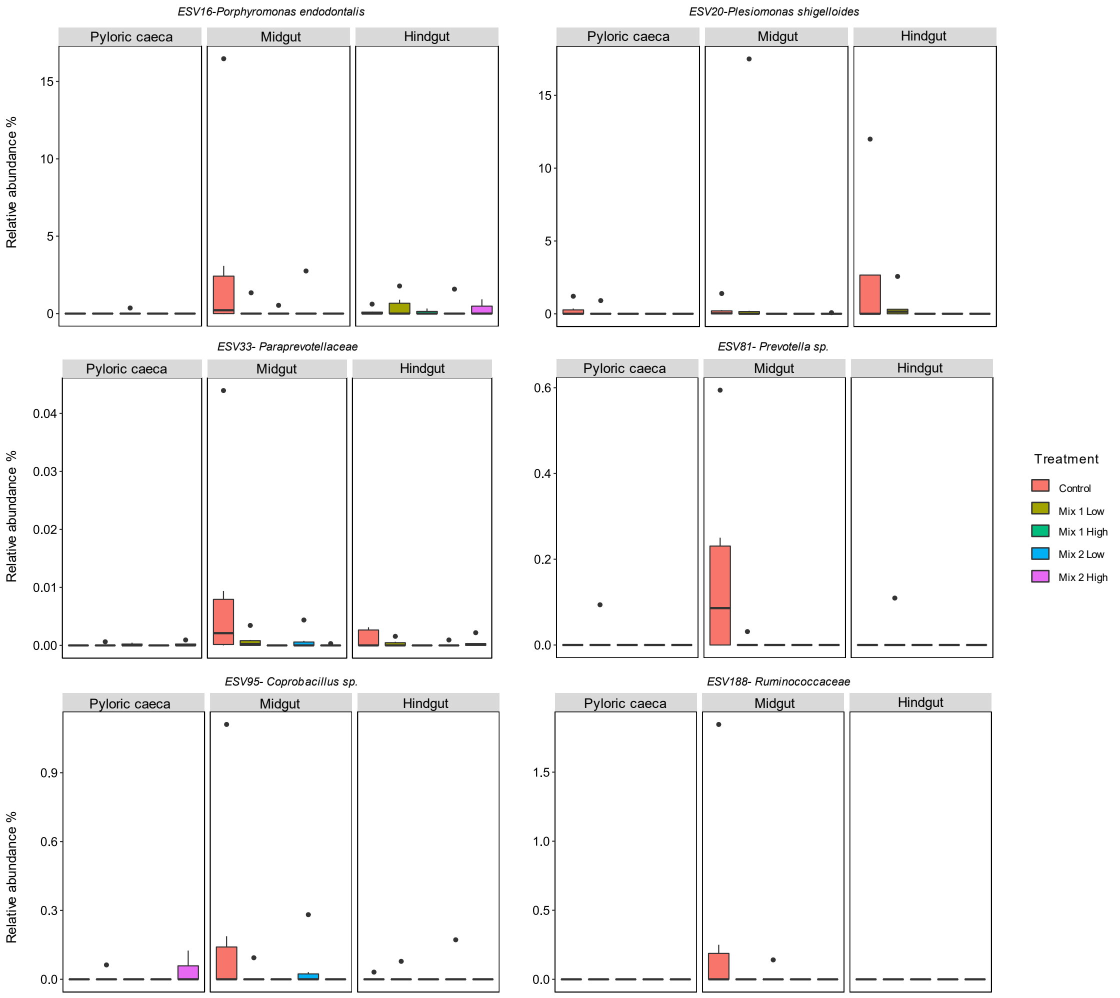

Supplementary Figure 11. Relative abundance of antibiotic-sensitive taxa across parts and diets. On the Y-axis is shown the relative abundance in % and on the X-axis the different dietary groups.

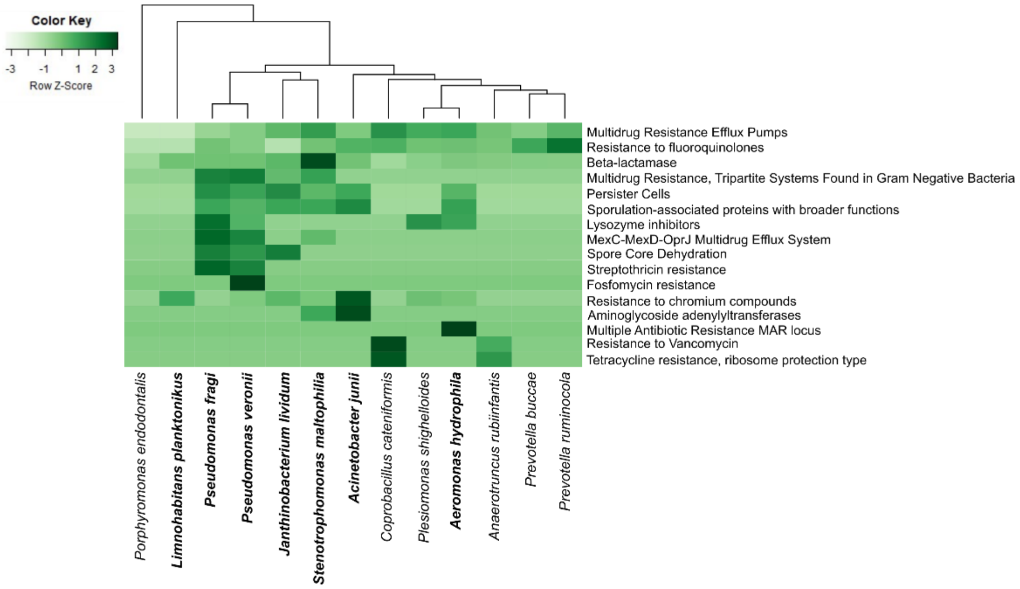

Supplementary Figure 12. Heatmap showing the antibiotic resistance gene abundance in the resilient (bold) and the antibiotic-affected microbes, showing that most of the persistent microbes cluster together. Antibiotic resistant genes were identified using the RAST server.
